# Status dystonicus resembling the intrathecal baclofen withdrawal syndrome: a case report and review of the literature

**DOI:** 10.1186/1752-1947-4-294

**Published:** 2010-08-31

**Authors:** William Muirhead, Ibrahim Jalloh, Michael Vloeberghs

**Affiliations:** 1 School of Medicine, University of Nottingham, Queens Medical Centre, Derby Road, Nottingham, NG7 2UH, UK; 2 Department of Neurosurgery, Queens Medical Centre, Nottingham University Hospitals, NHS Trust, Nottingham, NG7 2UH, UK

## Abstract

**Introduction:**

Status dystonicus is a rare but life-threatening disorder characterized by increasingly frequent and severe episodes of generalized dystonia that may occur in patients with primary or secondary dystonia. Painful and repetitive spasms interfere with respiration and may cause metabolic disturbances such as hyperpyrexia, dehydration, respiratory insufficiency, and acute renal failure secondary to rhabdomyolysis. Intrathecally administered baclofen, delivered by an implantable pump system, is widely used for the treatment of refractory spasticity. Abrupt cessation of intrathecal baclofen infusion has been associated with a severe withdrawal syndrome comprised of dystonia, autonomic dysfunction, hyperthermia, end-organ failure and sometimes death. The aetiology of this syndrome is not well understood. Status dystonicus describes the episodes of acute and life-threatening generalized dystonia, which occasionally manifest themselves in patients with dystonic syndromes.

**Case presentation:**

We present the case of a nine-year-old Caucasian boy who experienced a severe episode of status dystonicus with no known cause and clinical features resembling those described in intrathecal baclofen withdrawal. Our patient subsequently underwent the placement of an intrathecal baclofen pump without incident.

**Conclusion:**

The similarity between the clinical features of the case we present and those reported in connection to abrupt withdrawal of intrathecal baclofen is emphasized. Several drugs, although not intrathecal baclofen withdrawal, have previously been associated with status dystonicus. The similarity between the life-threatening dystonic episode experienced by our patient, and those reported in intrathecal baclofen withdrawal, highlights the possibility that, rather than representing a true physiological withdrawal syndrome, abrupt withdrawal of intrathecal baclofen may simply precipitate an episode of status dystonicus in susceptible individuals. The clinical similarities between the intrathecal baclofen withdrawal syndrome and status dystonicus have not previously been highlighted.

## Introduction

Status Dystonicus (SD) is a rare but life-threatening disorder characterized by increasingly frequent and severe episodes of generalized dystonia that may occur in patients with primary or secondary dystonia. Painful and repetitive spasms interfere with respiration and may cause metabolic disturbances such as hyperpyrexia, dehydration, respiratory insufficiency, and acute renal failure secondary to rhabdomyolysis. It is common for a single patient to experience multiple bouts of SD. Episodes of SD may be precipitated by infection, surgery, stress or changes in medication but frequently no cause is identified. A patient with SD is best managed with sedation and may require intubation and ventilation in severe cases [1].

Intrathecal baclofen (ITB) has been used extensively for the management of the spasticity that results from a number of spinal and supra-spinal neurological disorders. ITB is delivered directly to the intrathecal cerebrospinal fluid (CSF) via a variable rate implantable pump system, most commonly via a catheter placed in the lumbar spine; but thoracic and cervical levels can also be used. When compared with oral dosing, the intrathecal route enables much higher CSF concentrations with a diminished side-effect profile.

The most common complications observed with the use of ITB are related to infection, programming errors and catheter migration or fracture. Abrupt withdrawal from ITB has been associated with a constellation of clinical sequalae including pruritus, hyperthermia, confusion, and haemodynamic instability progressing to rhabdomyolysis, disseminated intravascular coagulation, multi-end organ failure and, ultimately, death [2-5]. Numerous reports are described in the literature and the term 'ITB withdrawal syndrome' has been used to define them. Comparisons have been drawn between ITB withdrawal syndrome and other acute, life-threatening systemic conditions such as neuroleptic malignant syndrome, malignant hyperthermia and serotonergic syndromes [6-8].

Whether the clinical features observed are the result of a true withdrawal syndrome representing physiological dependence is not clear. Often the diagnosis is made in the context of multiple co-morbidities, when treatment for other acute diagnoses, such as sepsis, has commenced. The ability to measure ITB concentrations is usually not available to give evidence to the proposed cause of the clinical episode.

We report the case of a patient experiencing an acute episode of SD with clinical features comparable to those described in ITB withdrawal. This patient has subsequently been fitted with an ITB pump. We compare the features of this patient's episode of SD with those of the previously reported paediatric cases of ITB withdrawal and discuss the implications for the diagnosis and significance of complications due to abrupt ITB withdrawal.

## Case presentation

A previously well nine-year-old Caucasian boy was admitted to the Paediatric Intensive Care Unit (PICU) after having a severe hypoxic brain injury following a near hanging. Recovery was complicated by recurrent, persistent dystonic extensor spasms. Magnetic resonance imaging (MRI) six days after admission showed widespread infarction in the watershed areas of both hemispheres and abnormal signal changes within the thalami. Bouts of spasticity were associated with tachycardia, pyrexia and hypoxia. An EEG did not demonstrate an association with cortical activity. Two weeks after admission our patient was discharged to the general ward, apyrexial and normotensive with normal inflammatory markers and measures of renal and hepatic function. His GCS was 5. His medications included lorazepam 1.3 mg PO/IV QDS, baclofen 20 mg PO TDS, chloral hydrate 1 g QDS and paracetamol 390 mg PO/PR QDS (his weight was 26 kg).

Five days after discharge from PICU he was noted to have a temperature of 39.5°C. Nystatin (100,000 QDS) was started for oral candida but no other source of infection was clinically identified. Blood cultures were negative and C-Reactive Protein (CRP) was 2.8 mg/L. Routine haematology and tests of renal and hepatic function were unremarkable apart from an elevation of aspartate transaminase (175 IU/L).

Our patient continued to deteriorate over the next 24 hours, becoming more agitated and experiencing episodes of haematemesis. Lorazepam was increased to 2 mg PO/IV QDS and chloral hydrate to 1.3 g QDS. Ranitidine (75 mg BD) was also started. He remained cardiovascularly stable (respiratory rate 20 rpm; heart rate 132 bpm; blood pressure 114/75 mmHg) but experienced progressive hypoxia and continued spasticity. Trihexyphenidyl (0.5 mg BD) was started.

Spasticity, fever (up to 40.4°C), and hypoxia (88% oxygen saturations) accompanied by haematemesis and diarrhoea continued over the next six days. The patient became tachycardic, tachypnoeic and hypotensive (respiratory rate 28 bpm; heart rate 190 bpm; blood pressure 110/40 mmHg) Chest radiographs excluded a chest infection and there was no evidence of urinary tract infection on urine analysis. There was still no elevation of CRP.

Eight days after the start of this episode the patient developed a cough and his CRP was found to be elevated (14.4 mg/L). Broad-spectrum antibiotics were started for a presumed lower respiratory tract infection. Extensor posturing and hyperpyrexia continued and the patient was noted as having spasmodic hypertensive episodes. His urine was also noted to be very dark and nine days after the start of the episode his serum creatine kinase (CK) was found to be 55,869 IU/L. His fluids were changed to a bicarbonate drip of 2 mmol/kg of NaHCO3 per day.

Over the next few days, our patient became apyrexial and episodes of dystonia became less frequent. Serum CK started to fall. Liver function tests, however, were noted to be deranged (Alanine aminotransferase 621 IU/L; Aspartate transaminase 411 IU/L; Albumin 30 g/L). Screening for inbuilt errors of metabolism revealed no abnormalities. The patient's condition steadily improved and, although he continued to have exacerbations of spasticity, he did not experience another dystonic crisis of similar severity. He was finally discharged from hospital nine months after his first admission. Seven months after his hypoxic injury, the patient underwent elective placement of a programmable pump device for continuous ITB with good result. At a five-month follow-up he weighed 35.9 kg with an intrathecal baclofen infusion of 100 micrograms per day. He was also prescribed Trihexyphenidyl 5 mg TDS but no oral baclofen. He continued to have a good response to the ITB.

## Discussion

Infection, surgery, stress and the introduction or withdrawal of drugs have previously been linked to precipitating SD, although often no precipitating factor is identified [9]. In our patient SD may have been precipitated by the introduction of any of the new medications that were started or a withdrawal episode due to emesis. However, one of the medications taken by this patient has previously been linked to SD. However, the introduction of benzodiazepines (although not specifically lorazepam) has been linked to SD. Withdrawal from long-term oral baclofen is less severe than withdrawal from intrathecal baclofen but has been linked to a SD-like syndrome of spasticity and fever. However, the fact that this patient had only been started on oral baclofen relatively recently and that emesis did not precede his agitation and pyrexia militate against oral baclofen withdrawal being the cause of status dystonicus in this case [10].

Rhabdomyolysis, as seen in our patient, is a common complication of SD, owing to intense muscle activity [11]. During admission, our patient's pyrexia was attributed to hypothalamic failure. However, pyrexia regularly occurs in SD [1,11] due to the massive exothermic effects of prolonged muscle contraction. Occasionally SD can lead to multi-organ failure and even death. Given that our patient subsequently had an ITB pump fitted, it is notable that the clinical features of his episode of SD were comparable to those that have been attributed to ITB withdrawal. A literature review identified nine previously reported episodes of ITB withdrawal in the paediatric population and they are summarized in Table 1.

**Table 1:** Summary of reported cases of ITB withdrawal

Author/Reference	Patient and Primary Diagnosis	Reason for ITB failure	Features Reported (those in italics were *not *a feature of the case we report)	Management and Outcome
Hansen *et al. *[12]	11 F Cerebral Palsy	Removal of infected ITB Pump several hours previously	Dystonia, Pyrexia, Rhabdomyolysis, Tachycardia, Hypertension, Reduced O2 Saturation, *Diaphoresis*	Admitted to ICU and continuously sedated. Oral baclofen, dantrium, cyproheptadine, propofol and clonidine. Pump replaced with good result.
Samson-Fang *et al. *[7]	9 M Cerebral Palsy	Disconnection of distal tubing from proximal segment. Time to onset unknown.	Dystonia, Pyrexia (39.5°C), Rhabdomyolysis (15,200 IU/L), Agitation, Hypertension, *Seizure*, *Scrotal and Ankle Oedema*, *Clotting Derangement*, *Metabolic Acidosis*, Transaminase Elevation, *Leukocytosis, Renal Failure*	Oral baclofen and clorazepate. Pump system revised. Patient subsequently deferevesced and tone returned to baseline.
Kao *et al. *[4]	4 M Cerebral Palsy	Pump noted to be empty and beeping. Time to onset unknown.	Dystonia, Pyrexia (42.1°C), Tachycardia, Tachypnoea, Hypertension, Reduced O2 Saturation, Wheeze, Crepitations, Leukocytosis	Admitted to PICU. Paracetamol and bronchodilators. Following pump refill, fever and tachycardia resolved over four to eight hours. Recovery.
Salazar *et al. *[13]	14 M Cerebral Palsy	Less than 1 mL left in pump reservoir	Dystonia, Rhabdomyolysis (2500 IU/L), Tachycardia, Agitation, Hypertension, *Diaphoresis*, Haematemesis, *Melaena*, *Clotting Derangement*, Transaminase Elevation	Pump refilled. Diazepam and cyproheptadine used to control tone. Discharged on hospital day four.
Saveika *et al. *[14]	14 F Cerebral Palsy	Pump Stall 8 hrs previously	Dystonia, Pyrexia (37.8°C), Tachycardia, Agitation, *Pruritis*	Oral baclofen and cyproheptadine. Recovery within 24 hours.
Alden *et al. *[5]	14 M Adrenoleukodystrophy	Catheter failure 1 week following spinal surgery	Dystonia, Pyrexia (40°C), Tachypnoea	IV lorazepam and oral baclofen. Pump and catheter revision. Discharged on post-operative day two.
Zuckerbraun *et al. *[15]	7 F Traumatic Brain Injury	Due for refill the following day	Dystonia, Pyrexia (40.8°C), Tachycardia, *Impaired Consciousness*, Tachypnoea, *Diaphoresis, Seizure, Leukocytosis*	Baclofen bolus and pump refill. Defervesced over four hours.
Zuckerbraun *et al. *[15]	14 M Traumatic Brain Injury	ITB catheter disconnection. Time to onset unknown.	Dystonia, Pyrexia (41°C), Tachycardia, Agitation, Hypotension	Four day admission. Misdiagnosed with viral illness and discharged. Withdrawal episode diagnosed on the basis of a subsequently identified catheter disconnection. Catheter subsequently revised with an improvement in tone.

The five most commonly reported clinical features of ITB withdrawal (dystonia, pyrexia, tachycardia, agitation, hypertension) all occurred in our patient. Rhabdomyolysis was a feature of three of the nine extant reports of complications attributed to ITB withdrawal.

The similarity between the case we report and the previously reported cases of complications attributed to ITB withdrawal raises two important points.

First, the presence of an ITB pump in a patient with this constellation of symptoms does not exclude the possibility that the patient is experiencing an episode of SD coincidentally. In one third of the cases reviewed, the time of the presumed causative system failure was unknown. ITB infusion systems frequently fail and it is possible that these had failed previously and that this was only identified during an unrelated subsequent episode of SD. Indeed, there is one case report in the adult literature in which no evidence for ITB pump failure is ever identified but "intermittent pump stall" is postulated solely on the basis of the patient's clinical presentation [8]. Caution should be exercised when attributing complications to ITB withdrawal in the absence of a direct temporal relationship between the onset of symptoms and ITB infusion system failure.

Second, the similarity between this episode of SD and the complications attributed to ITB highlights the possibility that the complications witnessed in episodes of ITB withdrawal may be instances of SD. Abrupt withdrawal of both lithium and tetrabenazine have previously been implicated in precipitating SD [9], and abrupt withdrawal of ITB may have a similar effect. If so, this would have significant implications both for the management of complications associated with ITB infusion and for the perceived risks associated with the placement of an ITB infusion system.

## Conclusion

We have presented a case of status dystonicus with features comparable to those attributed to ITB withdrawal; the case serves as a caution against over-diagnosing ITB withdrawal syndrome and highlights the possibility that, the complications observed in "ITB withdrawal syndrome," and exceptionally rarely associated with oral baclofen withdrawal, may themselves be an instance of status dystonicus precipitated by the drug's withdrawal.

## Competing interests

The authors declare that they have no competing interests.

## Consent

Written informed consent was obtained from the patient's guardian for publication of this case report and any accompanying images. A copy of the written consent is available for review by the Editor-in-Chief of this journal.

## Authors' contributions

WM reviewed the patient's clinical notes and wrote them up as a case report. WM and IJ jointly carried out the literature review included. IJ wrote a large part of the introduction of this report. MV wrote a substantial part of the conclusion/discussion of this report. All authors read and approved the manuscript.
